# Meta-analysis of the therapeutic effect of acupuncture on dysphagia in patients with Parkinson disease

**DOI:** 10.1097/MD.0000000000036698

**Published:** 2023-12-22

**Authors:** Liu Jiayu, Wu Minmin, Luwen Zhu

**Affiliations:** a Graduate School, Heilongjiang University of Traditional Chinese Medicine, Harbin, China; b The Second Affiliated Hospital of Heilongjiang University of Traditional Chinese Medicine, Harin, China.

**Keywords:** acupuncture, meta-analysis, Parkinson, swallowing function

## Abstract

**Objective:**

To systematically evaluate the therapeutic effect of acupuncture on dysphagia in patients with Parkinson disease (PD).

**Method:**

We searched CNKI, WF, VIP, CBM, Cochrane Library, and Web of Chinese Biomedical Literature Randomized controlled trials on the efficacy of acupuncture in the treatment of dysphagia in patients with PD was retrieved from Science, Embase, and PubMed databases from establishment to October 2022. Outcome indicators included clinical efficacy, swallowing function, hemoglobin, and serum albumin. Literature screening and data extraction of included literature were conducted independently by 2 reviewers, and literature quality was evaluated according to the standards of the Cochrane Collaboration network. Data analysis was performed using Review Manager 5.3 and Stata14.0 software.

**Results:**

466 patients were included in 7 literature, 234 in the observation, and 232 in the control groups. The results of the meta-analysis showed the clinical efficacy in the observation group [odd ratio = 0.25, 95% confidence interval (95%CI) (0.15, 0.40), *P* < .01]. Swallowing function [standardized mean difference (SMD) = −0.96, 95%CI (−1.24, −0.68), *P* < .01]; hemoglobin index level [SMD = −0.72, 95%CI (−1.25, −0.20), *P* < .01]; serum albumin index level [SMD = −1.25, 95%CI (−2.19, −0.31), *P* < .01].

**Conclusion:**

Acupuncture has a specific curative effect on dysphagia in patients with PD, and the therapeutic effect is more significant than that in the control group, which can improve the dysphagia function and nutrition level in patients with PD more effectively.

## 1. Introduction

Parkinson disease (PD), also known as palsy tremor, belongs to the range of “tremor syndrome” and “spasmodic syndrome” in traditional Chinese medicine. The disease is located in the meridians and can involve the liver, kidney, spleen, and other viscera. Its incidence increases with age, seriously jeopardizing the health of middle-aged and older people.^[1]^ In addition to the typical motor symptoms such as tremors and panicked gait, patients with PD are also accompanied by various non-motor symptoms. Swallowing disorder is one of the most common non-motor symptoms of PD, which can occur at any time during PD. These non-motor symptoms are easily confused with other conditions, which increases the chance of being ignored and dramatically reduces patients’ quality of life. Patients’ social interaction and participation experience will be affected, and the psychosocial burden will also increase.^[2]^

Dysphagia caused by PD is a more complex problem at present. Studies have shown that the incidence of dysphagia in patients with PD ranges from 18.5% to 82%.^[3]^ The condition is progressive, seriously affecting the intake of drugs and nutrients, leading to the decline of drug efficacy, the reduction of nutritional level, and then nutritional deficiency and dehydration, the most severe adverse factors for the prognosis of patients with PD.^[4]^ At this stage, the swallowing disorder of most patients with PD is mainly treated with drug therapy to assist in rehabilitation training. Still, the effect is not significant enough to prevent disease development.^[5]^ Studies have shown^[6]^ that acupuncture has a bidirectional regulatory effect, which can not only improve the symptoms of PD but also reduce the toxic and side effects of drugs. Combined with rehabilitation and drug treatment, acupuncture can play a synergistic role, thus improving the curative effect.

Acupuncture, as one of the treatment methods of traditional Chinese medicine, regulates zang-fu organs, relaxes tendons, and clears collaterals. Especially in recent years, acupuncture therapy has been widely used to treat swallowing disorders secondary to PD, stroke, brain stem injury, nasopharyngeal cancer, and other diseases.^[7]^ Li ^[8]^ stimulated the corresponding points on the meridians to make patients feel tingling and heaviness, further, promote the connection between the swallowing center and the surrounding organs, improve the excitability of dopaminergic neurons, significantly reduce the tension of muscle groups related to swallowing, and make up for the shortage of deep muscles of the tongue and pharynx that could not be affected by conventional swallowing training, thus improving the swallowing disorder of patients with PD. To achieve the purpose of delay and treatment.^[9]^

At present, the amount of clinical literature on acupuncture treatment of swallowing disorder in PD shows an increasing trend, which indicates that clinical and scientific researchers increasingly value this therapy. However, according to the relevant medical literature retrieved by the evidence-based medicine platform, this clinical observation literature needs to have appropriate systematic analysis and more high-quality studies are needed to prove their effectiveness. Therefore, it is necessary to conduct a meta-analysis on the therapeutic effect of acupuncture in the treatment of dysphagia in patients with PD, and evaluate its clinical efficacy, to provide evidence-based medical evidence for the clinical application of acupuncture in the treatment of dysphagia in PD.

## 2. Materials and methods

### 2.1. Study registration

The protocol of this review will be conducted and reported by the Preferred Reporting Items for Systematic Reviews and Meta-Analysis Protocols statement guidelines. The protocol has been registered on the International Prospective Register of Systematic Reviews (PROSPERO), and the trial registration number is CRD42023369730.

### 2.2. Search strategies

The search strategy will follow the Cochrane Handbook guidelines (5.1.0). From the beginning to April 2023, we will retrieve the following electronic databases: Cochrane Central Register of Controlled Trials (Central), PubMed, EMBASE, Web of Science, China National Knowledge Infrastructure, Chinese Biomedical Literature Database, And Wan Fang-Database. Meanwhile, clinical trial registries, like the WHO International Clinical Trial Registry Platform and Chinese Clinical Registry, will be searched for ongoing trials with unpublished data. Incomplete data will be obtained by contacting the corresponding authors. The retrieval strategy is adjusted according to the database using the combination of subject headings and free words. Taking PubMed as an example, the following keywords were used for the literature search: (((“Parkinson Disease” [Mesh]) OR (PD [Title/Abstract] OR Lewy Body PD [Title/Abstract]OR Paralysis Agitans [Title/Abstract] OR parkinsonism [Title/Abstract])) AND ((“acupuncture” [MeSH]) OR (Pharmacopuncture [Title/Abstract]))) AND ((deglutition disorders [MeSH Terms]) OR (Swallowing Disorders [Title/Abstract] OR Dysphagia[Title/Abstract] OR Disorders, Deglutition[Title/Abstract] OR Dysphagia[Title/Abstract] OR Oropharyngeal Dysphagia[Title/Abstract] OR Dysphagia, Oropharyngeal[Title/Abstract] OR Esophageal Dysphagia[Title/Abstract] OR Dysphagia, Esophageal[Title/Abstract])). For specific retrieval strategies, take PubMed as an example, as shown in Table 1.

**Table 1:** PubMed search strategy.

Process	Accession number
#1 #2 #3 #4 #5 #6 #7 #8 #9 #10	“Parkinson Disease” [Mesh] Parkinson Disease [Title/Abstract] OR Lewy Body Parkinson Disease [Title/Abstract]OR Paralysis Agitans [Title/Abstract] OR parkinsonism [Title/Abstract] #1 OR #2 “acupuncture” [MeSH] Pharmacopuncture [Title/Abstract] #4 OR #5 “Deglutition Disorder” [MeSH] Swallowing Disorders [Title/Abstract] OR Dysphagia[Title/Abstract] OR Disorders, Deglutition[Title/Abstract] OR Dysphagia[Title/Abstract] OR Oropharyngeal Dysphagia[Title/Abstract] OR Dysphagia, Oropharyngeal[Title/Abstract] OR Esophageal Dysphagia[Title/Abstract] OR Dysphagia, Esophageal[Title/Abstract] #7 OR #8 #3 AND #6 AND #9

### 2.3. Literature inclusion and exclusion criteria

#### 2.3.1. Inclusion criteria.

Study type: a randomized controlled trial (RCT) on the effect of acupuncture on swallowing function in patients with PD; study subjects: All patients met the diagnostic criteria for PD,^[10]^ were lucid, were able to cooperate with examination and treatment, and the swallowing function was evaluated by the Kota water test at grades II to V.^[11]^ Intervention measures: The control group received routine training (swallowing rehabilitation training, early swallowing training, oral sensorimotor training, anti-Parkinson drug therapy + swallowing rehabilitation therapy, rehabilitation training, functional training); the Observation group was treated with acupuncture therapy. Outcome indicators: clinical efficacy,^[12]^ swallowing function, hemoglobin (HB), serum albumin (ALB).

#### 2.3.2. Literature exclusion criteria.

Repeated publications; RCT studies such as reviews, conferences, patents, secondary analyses, and case reports; in addition to acupuncture, the observation group was also combined with other treatments different from the control group; dysphagia caused by other causes, such as cerebrovascular disease, trauma, neuromuscular disease, throat malignancies, digestive tract diseases, etc.

### 2.4. Literature selection and data extraction

The literature was searched and screened independently by 2 researchers. First, all the literature obtained in the preliminary search was imported into the NoteExpress software, and the software function was used to remove duplicate literature. Obviously, irrelevant literature was excluded by reading titles, abstracts, and keywords. Finally, the full text of the literature that may be included is read for further screening, the literature that does not meet the criteria is removed, and the literature to be included is obtained. The third party will check and decide again if the 2 researchers have different opinions. The extracted data mainly include author, publication year, sample size, patient age, intervention measures, course of treatment, and outcome indicators. If the information in the article needs to be completed, the corresponding author will be contacted.

### 2.5. Assessment of risk of bias in included studies

**Figure 1.**
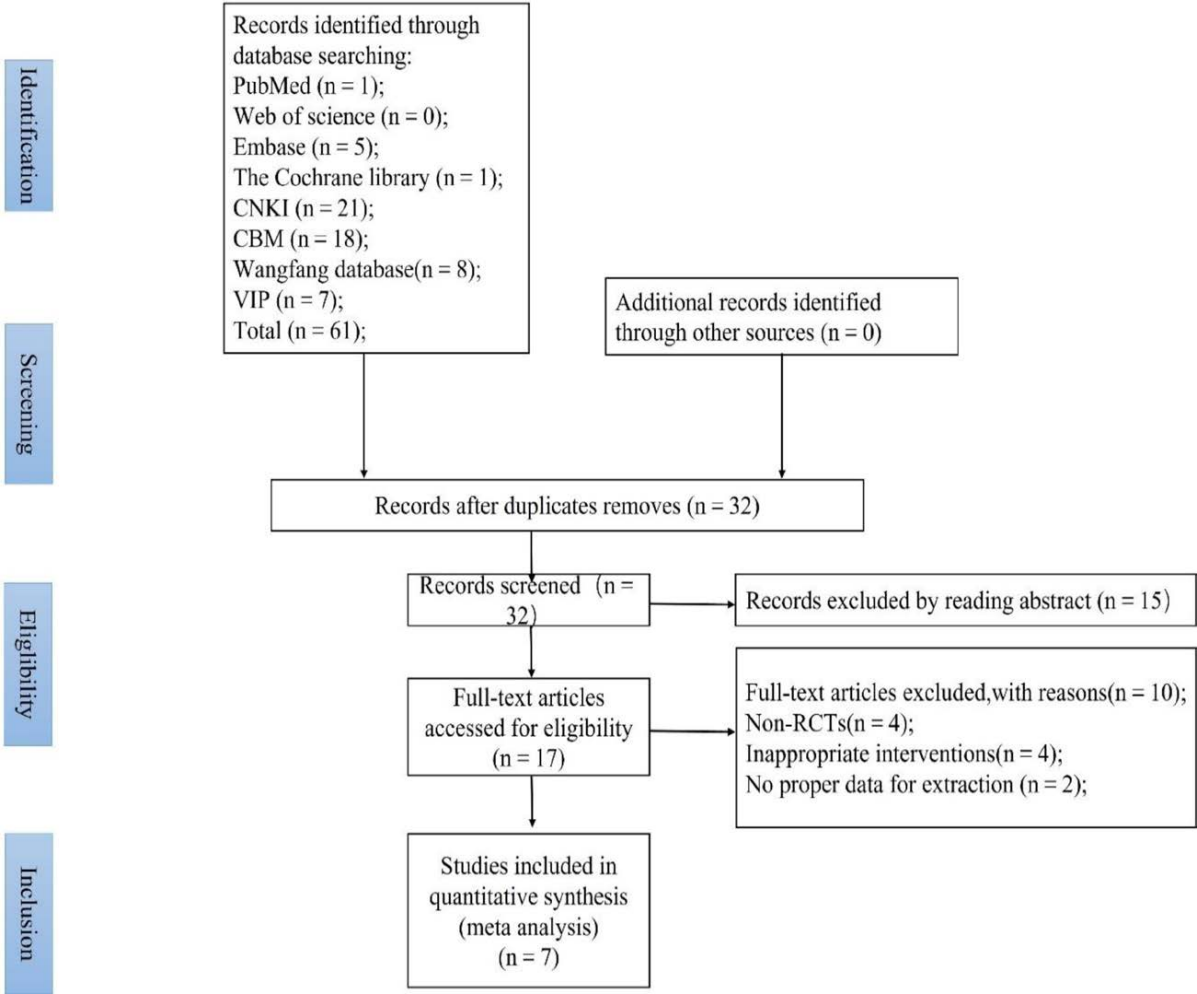
Risk ratio of literature bias.

**Figure 2.**
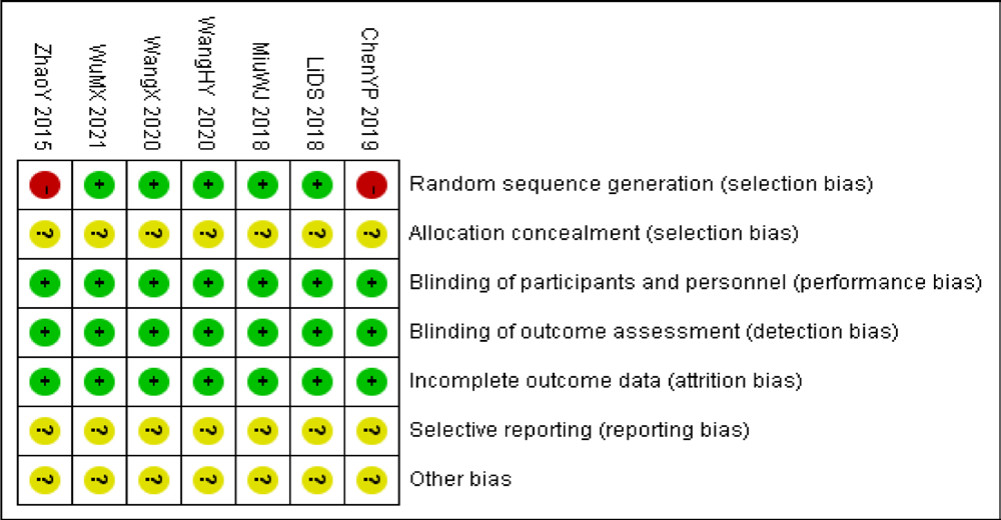
Risk ratio of literature bias.

The quality of the included literature was evaluated according to the risk bias assessment tool provided by Cochrane.^[13]^ The evaluation included random sequence generation, assignment concealment, blinding of subjects and measures, blinding of outcome evaluators, outcome data integrity, selective reporting, and other biases. According to the bias risk assessment criteria, the risk assessment of the included literature was carried out individually. The results were classified as “high risk,” “low risk,” or “unclear.” The bias risk bar chart and bias risk summary chart were drawn with the software Review Manager 5.3 (see Figs. 1 and 2).

First of all, from the point of view of evidence-based medicine, we need to collect research evidence for this topic as comprehensively as possible, so we searched all databases from the establishment of the database until October 2022. However, due to the reasons of intervention methods, most studies exist in China, and only 1 study by Wu Mingxia exists in PubMed. At the same time, we also searched this paper on CNKI and included this original paper. Secondly, we face up to this shortcoming. We also truthfully reported the search situation and search methods and strategies in the article, selected and excluded the articles that did not meet the inclusion criteria, evaluated the methodological quality of the study as a matter of fact, and explained the existing bias and impact on the results, hoping to attract the attention of clinical and scientific researchers.

### 2.6. Statistical method

The software Review Manager 5.3 and Stata14.0 were used for statistical analysis. The odd ratio (OR) and 95% confidence interval (95%CI) were used as the combined effect size indicators for taxonomic variables, and standardized mean difference (SMD) was used for continuous variables. SMD and 95%CI as pooled effect size indicators. The *Q* test and I^2^ were used to assess the heterogeneity among the studies. For included studies with no or low heterogeneity (I^2^ < 50%, *P* > .1), fixed-effect models were used for analysis. If the heterogeneity of included studies was high (I^2^ ≥ 50%, *P* < .1), the random effects model (REM) was used for analysis. Subgroups analyzed the factors that may lead to heterogeneity. A funnel plot was drawn by Stata14.0 software, and publication bias was analyzed. Whether there is, the *P* value represents statistical significance. When *P* < .05, the difference is considered significant; otherwise, there is no considerable significance.

## 3. Results

### 3.1. Article search and screening

**Figure 3.**
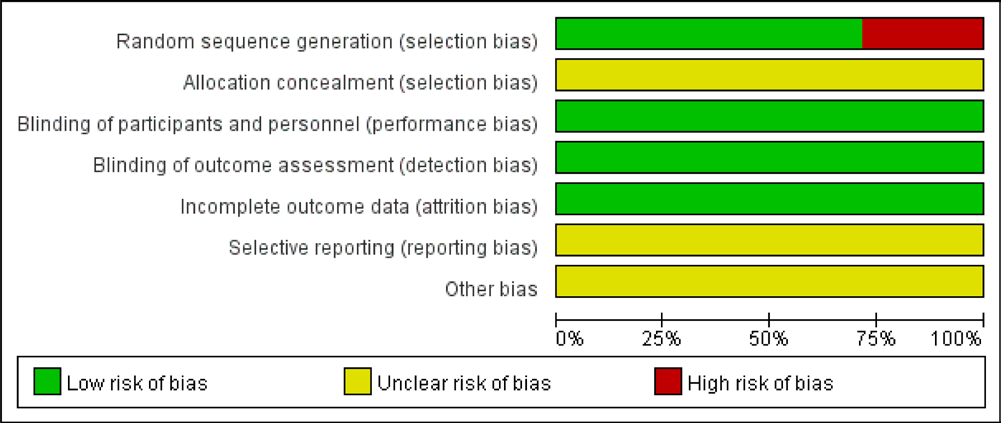
Literature screening flow chart.

According to the retrieval strategy, a total of 61 articles were retrieved, including 21 papers in CNKI, 7 pieces in VIP, 8 articles in Wan Fang Database, 1 article in PubMed, 1 article in Cochrane Library, 5 articles in Embase and 18 articles in Chinese Biomedical Literature Database. Importing Note Express 3.5.0 software to remove duplicate reports, 32 articles were initially retrieved. After reading the title and abstract, 15 review articles were eliminated. After reading the full text of the remaining 17 articles, 10 pieces were destroyed, including 6 non-RCT articles and 4 non-acupuncture-based articles in the observation group; finally, 7 articles were included, all in Chinese (Fig. 3).

### 3.2. Basic features included in the study

In this meta-analysis, 61 kinds of literature were initially examined, and 32 were left after duplicate literature was excluded. The titles, abstracts, and full-text reviews were excluded; conferences, patents, secondary analyses, case reports, non-RCT, and other literature that did not meet the inclusion criteria were further read. Finally, 7 RCTs^[8,14–19]^ were included, all of which were Chinese literature. The included publication period was 2011 to 2021; all were RCT. A total of 466 patients, ranging in age from 36 to 81 years, had a PD course of 0.3 to 14 years, including 234 in the observation group and 232 in the control group. In 4 papers^[15–18]^ literature, the intervention measures were acupuncture with a millimeter needle. In the paper,^[8]^ electroacupuncture was used as the intervention measure; the intervention measures of 2 literature^[14,19]^ were warm acupuncture and moxibustion. The essential characteristics of the included literature are shown in Table 2.

**Table 2:** Basic features of included studies.

Included research	Number of examples		Average age (yr)		Intervention		Acupuncture intervention (type; course of treatment; wk)	Observation metrics
	T	C	T	C	T	C		
Chen YP 2019^[19]^	40	40	53.6 ± 9.4	54.1 ± 9.5	Functional training + warm acupuncture	Functional training	Millimeter needle acupuncture Insert the moxibustion strip into the	①③
							end of the needle after getting qi	④
							Treatment time not mentioned	
Li DS 2018^[8]^	43	43	59.37 ± 4.89	58.46 ± 4.32	Early swallowing training + electroacupuncture	Early swallowing training	Electroacupuncture Once a d, 5 d a wk; 4 wk	①② ③④
Miu WJ 2018^[17]^	28	28	68.25 ± 7.33	67.50 ± 9.70	Swallowing rehabilitation training + acupuncture	Swallowing rehabilitation training	Millimeter needle acupuncture; once a d; 5 d a wk;4 wk	①③ ④
Wang HY 2019^[16]^	45	45	59 ± 10	59 ± 10	Oral sensory motor training + acupuncture	Oral sensory motor training	Millimeter needle acupuncture + tongue needle acupuncture	①②
							Once a d, 5 d a wk; 4 wk	
Wang X 2020^[15]^	20	20	63	64	Swallowing rehabilitation training + three needles for pharynx and 8 needles for antifibrillation	Swallowing rehabilitation training	Millimeter needle acupuncture; once a d; 10 d a course of treatment; 3–4 wk	①②
Wu MX 2021^[14]^	28	28	63 ± 10	65 ± 7	Swallowing rehabilitation training + acupuncture	Swallowing rehabilitation	Millimeter needle acupuncture + tongue needle acupuncture	①③
							Once a d, 5 d a wk	④
							6 wk	
Zhao Y 2015^[18]^	30	28	–	–	Rehabilitation training + warm acupuncture	Rehabilitation training	Millimeter needle acupuncture Insert the moxibustion strip into	①
							The end of the needle after getting qi	
							Treatment time not mentioned	

① Clinical efficacy, ② swallowing function, ③Hb, ④ALB “–” indicates that data cannot be obtained.

### 3.3. Methodological quality assessment

The Cochrane Manual evaluated the included literature in this study. A total of 7 kinds of literature were included, all of which were Chinese literature, of which 4^[8,14–16]^ were grouped by random number table method, 1 paper^[17]^ mentioned the randomization method but did not describe it in detail, and 2 papers^[18,19]^ randomized control methods were high-risk. None of the included literature mentioned assignment hiding and double blindness. All study data were complete, and no other bias was noted. The quality evaluation of the included literature is shown in Figure 1.

According to the revised Jadad scale, the quality of the included literature was evaluated with a score of 1 to 3 as low quality and 4 to 7 as high quality (Table 3).

**Table 3:** Literature quality evaluation of revised Jadad scale.

	Random sequence generation	Allocation concealment	Blinding	Withdrawal	Total score	Article quality
Chen YP 2019^[14]^	0	0	0	1	1	Low quality
Li DS 2018^[8]^	2	1	0	1	4	High quality
Miu WJ 2018^[15]^	1	0	0	1	2	Low quality
Wang HY 2019^[16]^	2	1	0	1	4	High quality
Wang X 2020^[17]^	2	1	0	0	3	Low quality
Wu MX 2021^[18]^	2	1	0	1	4	High quality
Zhao Y 2015^[19]^	0	0	0	2	2	Low quality

### 3.4. Methodological quality assessment

#### 3.4.1. Clinical efficacy.

**Figure 4.**
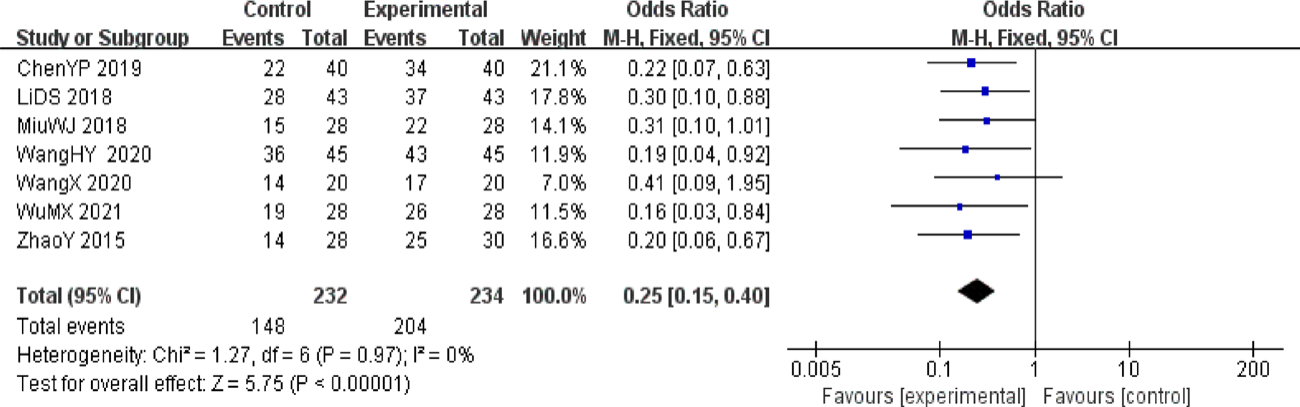
Forest map of clinical efficacy meta-analysis.

Clinical efficacy was reported in 7 papers.^[8,14–19]^ Heterogeneity test results suggested that there was homogeneity in multiple studies (*P* = .97 I^2^ = 0%), so a fixed effect model was used to calculate the combined effect size. The results showed that the clinical efficacy of the observation group was significantly better than that of the control group. The difference was statistically significant [OR = 0.25, 95%CI (0.15, 0.40), *P* < .01], indicating that acupuncture had a significant effect on the clinical efficacy of the treatment of dysphagia in patients with PD (Fig. 4).

#### 3.4.2. Swallowing function.

**Figure 5.**
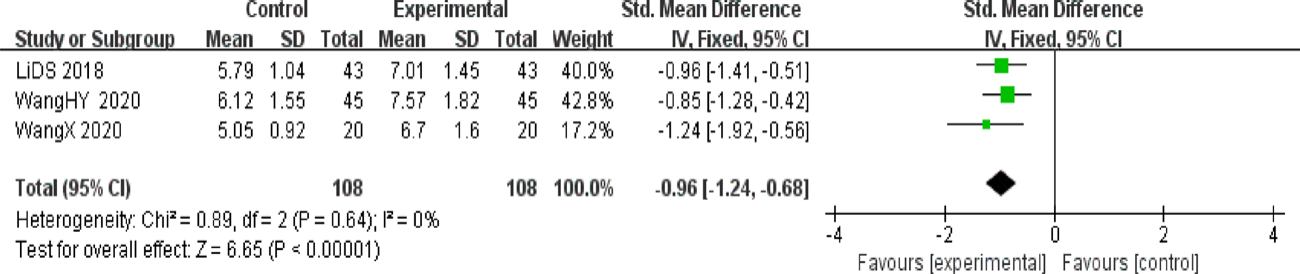
Forest map of meta-analysis of swallowing function.

The swallowing function was reported in 3 kinds of literature,^[8,16,17]^ and the heterogeneity test results indicated that the multiple studies were homogeneous (*P* = .64 I^2^ = 0%), so a fixed effect model was used for meta-analysis. The results showed that the improvement of swallowing ability in the observation group was significantly better than that in the control group. The difference was statistically significant [SMD = −0.96, 95%CI (−1.24, −0.68), *P* < .01], indicating that acupuncture had a substantial effect on swallowing ability in patients with PD (Fig. 5).

#### 3.4.3. Hemoglobin.

**Figure 6.**
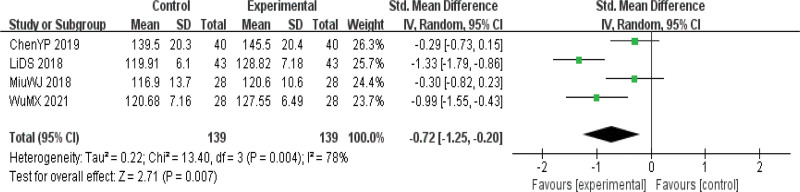
Forest map of hemoglobin (HB) index meta-analysis.

Four papers^[8,14,15,18]^ reported HB indexes, and the heterogeneity test indicated heterogeneity among the studies (*P* = .01 I^2^ = 78%), so the REM was used to calculate the combined effect size. The final results showed that the improvement of HB indexes in the observation group was significantly better than in the control group. The difference was statistically significant [SMD = −0.72, 95%CI (−1.25, −0.20), *P* < .01], indicating that acupuncture had a significant effect on the improvement of nutritional indexes in the treatment of PD (Fig. 6).

**Figure 7.**
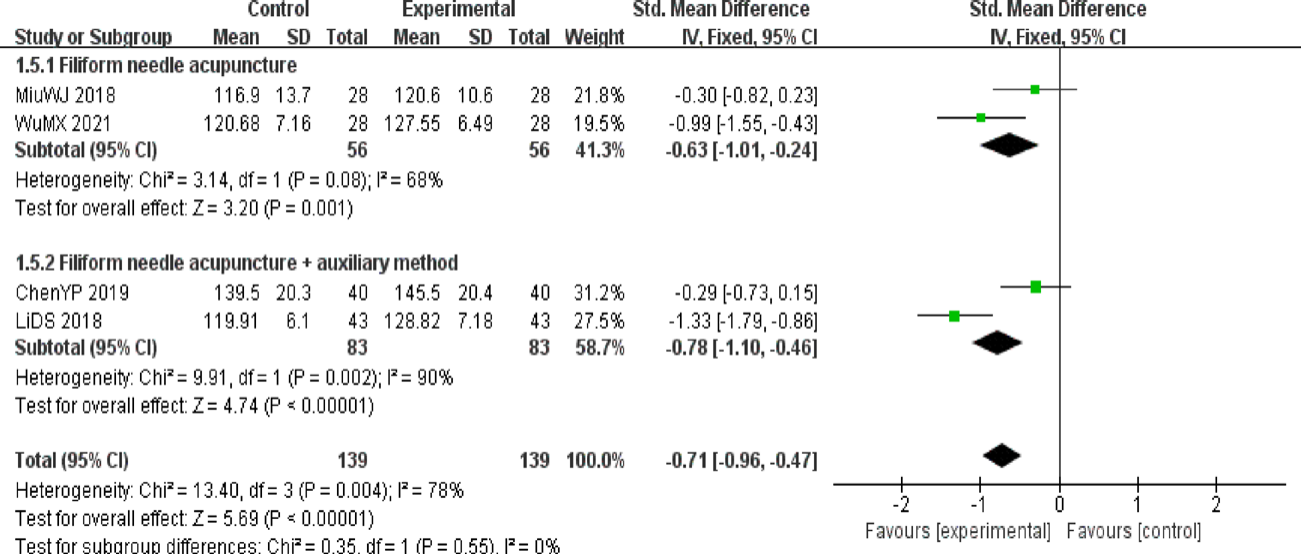
Forest map for subgroup analysis of hemoglobin (HB) indicators (interventions).

Subgroup analysis results: To explore the heterogeneity of this outcome indicator, subgroup analysis was conducted according to different interventions and the quality of the original literature. Two RCTs^[15,18]^ reported the effect of pure acupuncture on the HB index of PD patients, and the results showed that the indicators of both groups were increased after treatment, and the observation group was significantly better than the control group. The difference was statistically significant [SMD = −0.63, 95%CI (−1.01, −0.24), *P *< .01]. Two RCTs^[8,14]^ reported the effects of acupuncture plus adjuvant methods on HB indexes in patients with PD. The results showed that the indexes in both groups were increased after treatment, and the observation group was significantly better than the control group, with statistical significance [SMD = −0.78, 95%CI (−1.10, −0.46), *P* < .01]. The results of subgroup analysis according to different intervention measures showed that the heterogeneity of acupuncture, acupuncture, plus auxiliary methods was still significant, and acupuncture had no effect on HB indexes in PD patients. Subgroup analysis showed that acupuncture did not influence the HB index of PD patients by acupuncture type and quality of original literature. No source of heterogeneity was found in the 2 subgroups (Fig. 7).

#### 3.4.4. Serum albumin.

**Figure 8.**
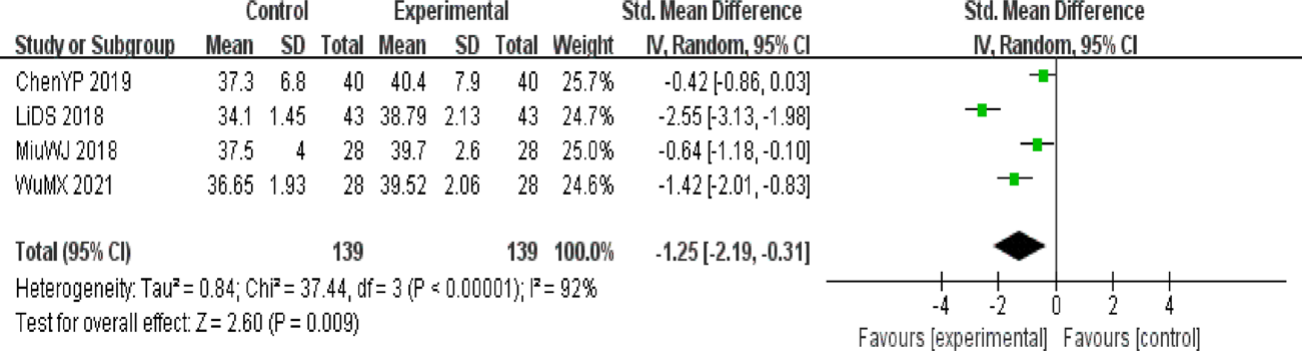
Forest map of serum albumin (ALB) index meta-analysis.

ALB indexes were reported in 4 kinds of literature,^[8,14,15,18]^ and the heterogeneity test results suggested that there was heterogeneity among the studies (*P* = .01 I^2^ = 92%), so the REM was used to calculate the combined effect size. The final results showed that the improvement of ALB indexes in the observation group was significantly better than that in the control group, with statistical significance [SMD = −1.25, 95%CI (−2.19, −0.31), *P* < .01], indicating that acupuncture had a significant effect on the improvement of nutritional indexes in the treatment of dysphagia in patients with PD.(Fig. 8).

**Figure 9.**
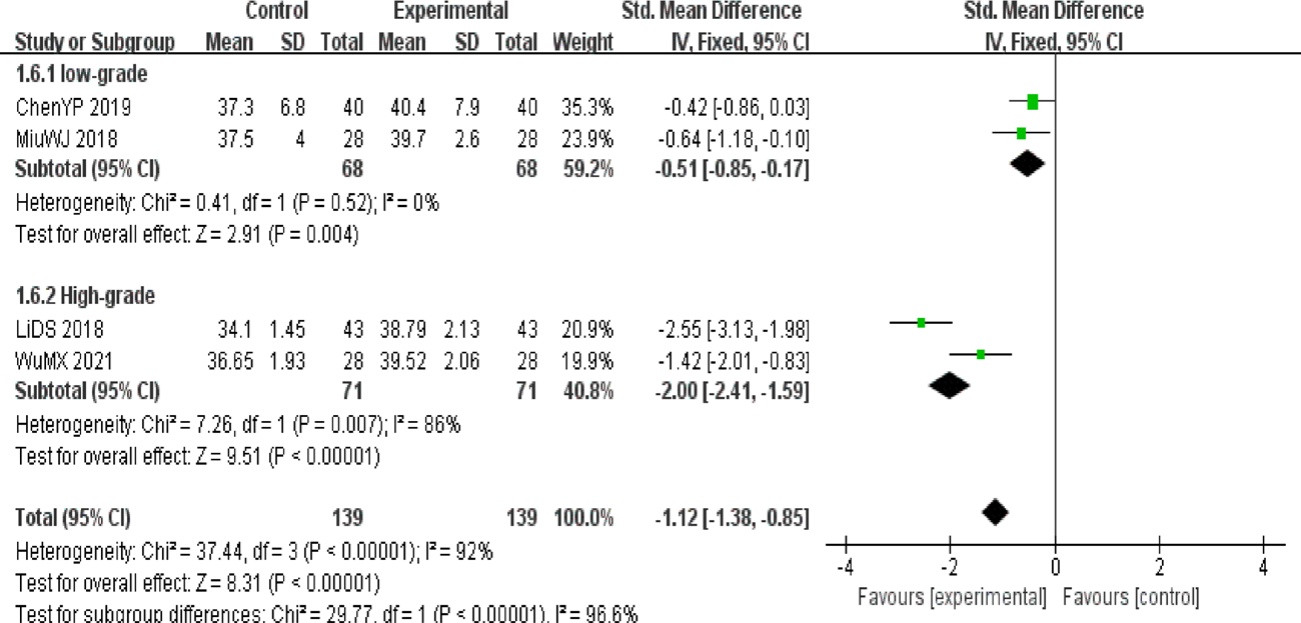
Forest map of subgroup analysis of serum albumin (ALB) index (quality of original literature).

Subgroup analysis results: To explore the heterogeneity of this outcome indicator, subgroup analysis was conducted according to different interventions and the quality of the original literature. Two RCTs^[8,18]^ reported the effects of high-quality actual literature acupuncture on ALB indexes of PD patients, and the results showed that the indexes of both groups were increased after treatment, and the observation group was significantly better than the control group. The difference was statistically significant [SMD = −2.00, 95%CI (−2.41, −1.59), *P *< .01]. Two RCTs^[14,15]^ reported the effect of low-quality original literature acupuncture on ALB indexes of PD patients. The results showed that the indexes of both groups were increased after treatment. The observation group was significantly better than the control group; the difference was statistically significant [SMD = −0.51, 95%CI (−0.85, −0.17), *P* < .01]. The results of subgroup analysis according to the quality of the original literature showed that the heterogeneity of high-quality and low-quality initial literature studies was still significant, and the HB index of PD patients was not affected by the quality of the original literature. The results of the subgroup analysis showed that acupuncture was not affected by the type of acupuncture and the quality of original literature on ALB indexes in PD patients. No source of heterogeneity was found in the 2 subgroups (see Table 4 and Fig. 9).

**Table 4:** Subgroup analysis results of HB and ALB.

Categorical regulating variable	k	d	95%CI	QB	The *P* value of QB
HB Types of HB intervention Filiform needle acupuncture Filiform needle acupuncture+auxiliary method Quality of original document Low quality High quality ALB Types of ALB intervention Filiform needle Acupuncture filiform needle acupuncture+auxiliary method Quality of original document Low quality High quality	2 2 2 2 2 2 2 2	−0.63 −0.78 −0.29 −1.19 −1.02 −1.48 −0.51 −2.00	[−1.01, −0.24] [−1.10, −0.46] [−0.63, 0.04] [−1.55, −0.83] [−1.78, −0.26] [−3.57, 0.62] [−0.85, −0.17] [−1.38, −0.85]	0.16 12.60 0.35 29.77	.69 .0004 .55 .01

k is the sample size, d is the effect size, and QB is the inter-group heterogeneity.

ALB = serum albumin, 95%CI = 95% confidence interval, HB = hemoglobin.

### 3.5. Sensitivity analysis

**Figure 10.**
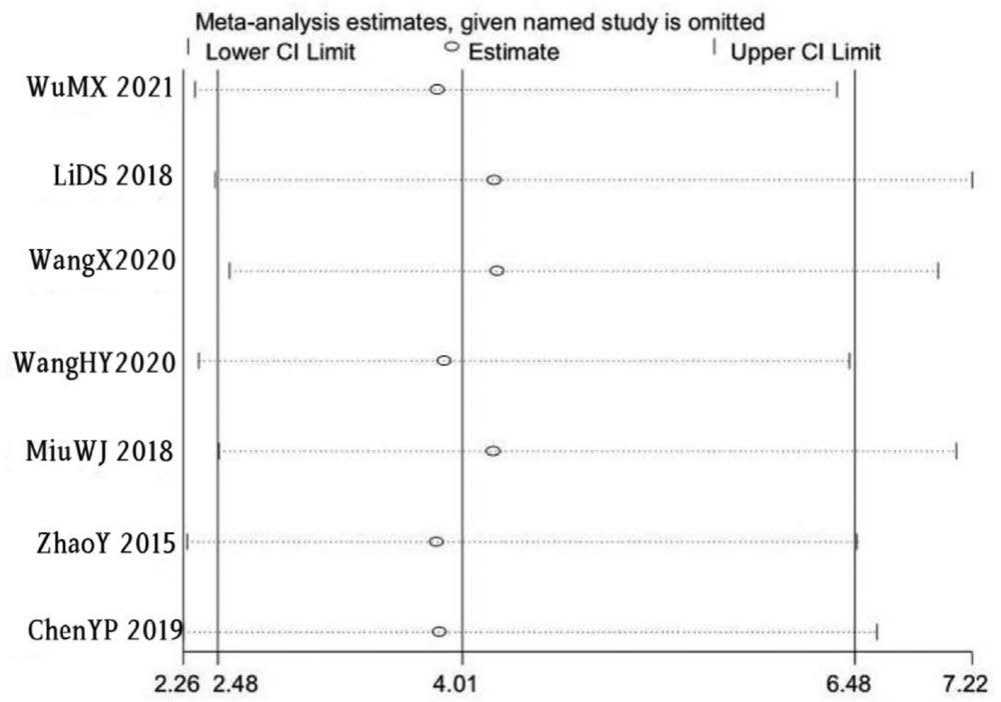
Sensitivity analysis diagram of included studies.

A meta-analysis was conducted by excluding individual references one by one. Through sensitivity analysis of the impact of a single study on the combined OR value, it was found that there was no significant change in OR and 95%CI, and all study point values fell in the 95%CI of the final result. The impact of excluding any item on the result would not exceed the CI, indicating that the meta-analysis results in this study were robust (see Fig. 10).

### 3.6. Assessment for reporting biases.

**Figure 11.**
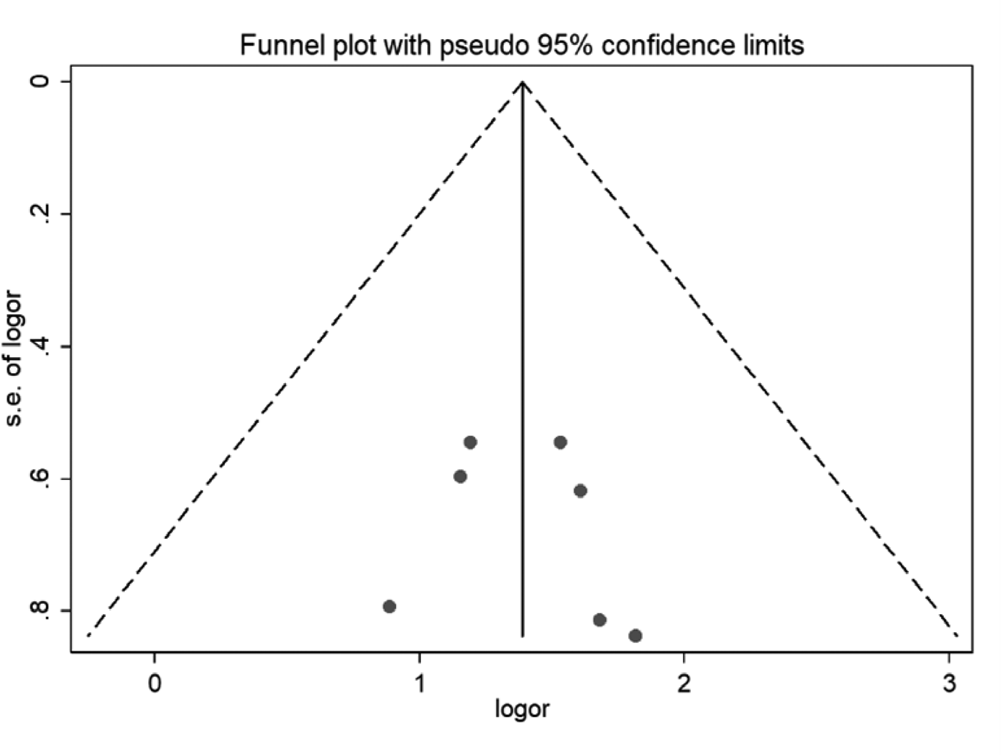
Publication bias funnel plot of included studies.

Publication bias analysis was conducted according to clinical efficacy, the main outcome index of the included literatures, and a funnel plot was drawn with the logarithm of the odds ratio OR of each study as the horizontal coordinate and the standard error of the logarithm of OR as the vertical coordinate. The funnel plot shows that the studies are evenly distributed on both sides and have good symmetry, suggesting that the possibility of publication bias is small (see Fig. 11).

**Figure 12.**
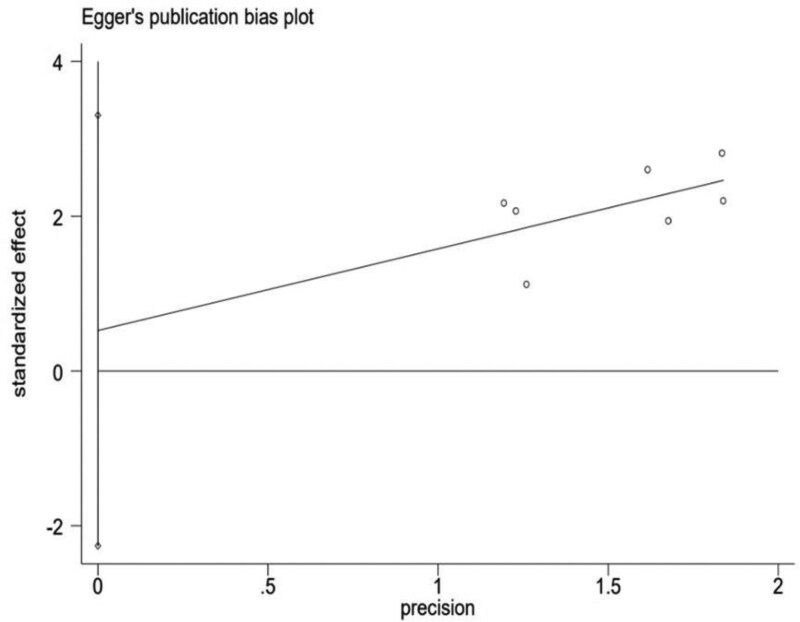
Egger graph of publication bias in included studies.

Since the funnel diagram may have subjective evaluation, Egger test is used to further detect the symmetry of the funnel diagram. The results show that there is no publication bias (Egger test, t = 1.51, *P* = .192 > .05), which indicates the stability of the results (see Fig. 12).

## 4. Conclusion

Presently, domestic studies focus on dysphagia secondary to cerebrovascular disease, and few studies pay attention to the relationship between dysphagia and dysphagia in patients with PD. However, dysphagia and dysphagia in PD are not uncommon in clinical practice.^[20]^ In the past 15 years, studies on the prevalence of swallowing disorders in patients with PD have confirmed that the vast majority of patients with PD will have swallowing disorders during the condition.^[21]^ It can seriously affect the quality of life and the ability of daily living of patients and even affect the motor symptoms of PD, which leads to the exacerbation of the disease. Therefore, how to improve the swallowing function of patients with PD has important clinical significance.

This study conducted a meta-analysis of 7 kinds of literature^[8,14–19]^ that used acupuncture therapy, all of which took clinical efficacy as the outcome index. The Kuwada drinking water test,^[22]^ proposed by Japanese scholar Toshio Kuwada was used to evaluate the treatment results and observe the total effective rate. The actual clinical effective rate is the sum of the clinical recovery rate, significant efficiency, and effective rate of patients with swallowing disorder of PD. It is a commonly used indicator in modern TCM clinical research.^[23]^ The results of the meta-analysis showed that the 95%CI horizontal line of the total effective rate of acupuncture in treating swallowing disorder in PD fell on the left side of the ineffective line, and the overall effect size difference was statistically significant. Compared with routine training alone, the clinical efficiency of acupuncture is significantly better than that of no treatment or rehabilitation treatment only. Therefore, clinical attention should be paid to the combination of acupuncture and routine training to improve the clinical efficacy of patients.

In 3 literatures,^[8,15,16]^ swallowing function was used as the outcome index, and the dysphagia evaluation scale (VFSS) was used to evaluate the swallowing function of patients before and after acupuncture treatment. Compared with the control group, the clinical efficacy and swallowing function of patients in the observation group were significantly better than those in the control group. It can improve the grade of VFSS and potable water tests. Li et al ^[8]^ pointed out that electroacupuncture can compensate for the lack of conventional swallowing training in the depths of the glossopharynx. Electroacupuncture is a treatment method that combines traditional acupuncture with current to expand the scope of acupuncture treatment, increase the amount of stimulation, improve muscle amplitude and reduce swallowing time, providing strong evidence for the treatment of swallowing disorders by acupuncture.^[7,24]^ The reason for the improvement of patients’ swallowing function is that acupuncture points can strengthen the coordination function of muscle movements related to swallowing and the flexibility of swallowing reflex and prevent disuse muscle atrophy and pulmonary complications in the pharyngeal muscle group.^[25]^ It has a good effect on the recovery of swallowing function, the reconstruction of neural pathways, and the self-care ability of patients’ daily life.^[26,27]^

According to studies, 67% of patients have insufficient dietary and nutritional intake, and the serological nutritional indexes HB and ALB are lower than those of healthy people.^[28]^ In 4 kinds of literature,^[8,14,17,19]^ the therapeutic effect of acupuncture on dysphagia caused by PD was measured by comparing serological nutritional indexes HB and ALB. There was no statistically significant difference between the dietary indexes of the 2 groups before treatment in the observation group, and the healthy indexes of the 2 groups were comparable, and the indexes of both groups increased after treatment. The observation group was significantly better than the control group (*P* < .05), suggesting that acupuncture greatly affected Parkinson patients with dysphagia and was better than only early swallowing training. With the improvement of swallowing function, the nutritional level of patients was also improved. In addition to the adjuvant therapy of drugs or swallowing rehabilitation, acupuncture can effectively improve the problems such as increased energy consumption, decreased energy intake, and impaired absorption caused by dopamine shortage,^[29]^ which can be used as one of the effective treatment methods to improve the swallowing function and nutritional level of patients with swallowing disorders in PD. Subsequent basic or clinical studies with larger samples are also needed to explore the regulatory effects and mechanisms of acupuncture on the swallowing nervous system in patients with PD swallowing disorder.

This study further conducted subgroup and sensitivity analyses because of the significant heterogeneity in the literature related to serological nutritional indicators. Subgroup analysis results showed that acupuncture point selection, acupuncture time, acupuncture frequency, drug type, dose, and drug frequency in the observation group were different in the 4 kinds of literature involving HB and ALB indexes,^[8,14,17,19]^ suggesting that the differences in intervention measures of patients in the observation group may be the source of heterogeneity. After removing the included kinds of literature one by one, the remaining types of literature were combined, and it was found that there was no significant change in I^2^ value and *P* value, indicating low sensitivity and robust meta-analysis results. At the same time, the article quality was also analyzed, and the results showed that the level of HB and ALB indexes was not affected by the article quality. However, due to the specificity of acupuncture treatment, it is difficult to blind the practitioner and the patient in clinical trials. The Chinese literature included in this study did not blind the evaluators, which led to exaggerated rehabilitation effects and other publication biases. Some studies showed that the observation group received swallowing rehabilitation training and drug therapy at the same time as acupuncture treatment, and the influence of rehabilitation training on treatment results cannot be ruled out. The above analysis can be the source of heterogeneity of serological nutritional indexes.

Although this study was strictly based on inclusion and exclusion criteria to screen relevant literature for meta-analysis, there are still certain limitations. Due to these factors, acupuncture method, point selection, course of treatment, use or not of electroacupuncture, and frequency selection, clinical and methodological heterogeneity may still be interfered with. Although the control group adopted the routine training mentioned in the article, it was also composed of different rehabilitation treatment methods, such as swallowing rehabilitation training, early swallowing training, oral sensorimotor training, anti-Parkinson drug therapy + swallowing rehabilitation therapy, rehabilitation training, and functional training. The differences of the control group may have certain effects on the effect indicators, but due to limited research, the control group is not able to perform the following functions: Subsequently, we will also classify and explain the routine training of the control group to explore its impact on the effect indicators. Further studies are needed to explore the efficacy of different intervention measures on swallowing disorders in patients with PD to explore the best intervention time for acupuncture treatment of swallowing disorders after PD, strive to further improve the clinical efficacy of acupuncture treatment, and provide better acupuncture point combination for patients with swallowing disorders in PD. Secondly, from the literature search results, most of the included research objects come from China, and the results may be affected by the patient population. To avoid bias caused by small sample studies, it is necessary and feasible to carry out extensive and multi-center studies in the future. Since most current studies focus on the short-term efficacy of acupuncture treatment and lack monitoring of the long-term development of the disease, future studies should pay attention to the long-term effectiveness of patients receiving acupuncture treatment and achieve long-term tracking.

In summary, acupuncture has a specific effect on treating swallowing disorders caused by PD. Due to the improvement of swallowing function, the nutritional level is also improved, and it plays an irreplaceable role in enhancing the living status and quality of life of patients with PD. Swallowing disorders caused by PD should be as widely regarded as swallowing disorders caused by cerebrovascular disease, and acupuncture treatment should be done as soon as possible. Future studies will also pay more attention to the therapeutic effect of different acupuncture methods and other treatment methods on swallowing disorders caused by PD, providing evidence support for further research and promotion of the therapeutic effect of acupuncture on swallowing disorders in patients with PD.

## Author contributions

**Data curation:** Wu Minmin.

**Formal analysis:** Wu Minmin.

**Funding acquisition:** Luwen Zhu.

**Investigation:** Liu Jiayu.

**Methodology:** Liu Jiayu.

**Resources:** Wu Minmin.

**Software:** Wu Minmin.

**Validation:** Luwen Zhu.

**Visualization:** Luwen Zhu.

**Writing – original draft:** Liu Jiayu.

**Writing – review & editing:** Liu Jiayu.
